# The clinical and genetic heterogeneity analysis of five families with primary periodic paralysis

**DOI:** 10.1080/19336950.2020.1857980

**Published:** 2020-12-21

**Authors:** Quanquan Wang, Zhe Zhao, Hongrui Shen, Qi Bing, Nan Li, Jing Hu

**Affiliations:** Department of Neuromuscular Disease, The Third Hospital of Hebei Medical University, Shijiazhuang, Hebei, China

**Keywords:** Primary periodic paralysis, Andersen-Tawil syndrome, *CACNA1S*, *SCN4A*, *KCNJ2*

## Abstract

To explore the clinical and genetic characteristics of five families with primary periodic paralysis (PPP). We reviewed clinical manifestations, laboratory results, electrocardiogram, electromyography, muscle biopsy, and genetic analysis from five families with PPP. Five families with PPP included: hypokalemic periodic paralysis type 1 (HypoPP1, *CACNA1S*, 1/5), hypokalemic periodic paralysis type 2 (HypoPP2, *SCN4A*, 2/5), normokalemic periodic paralysis (NormoPP, *SCN4A*, 1/5), and Andersen-Tawil syndrome (ATS, *KCNJ2*, 1/5). The basic clinical manifestations of five families were consistent with PPP, presenting with paroxysmal muscle weakness, with or without abnormal serum potassium. ATS was accompanied by ventricular arrhythmias, and skeletal and craniofacial anomalies, developing with a permanent fixed myopathy later. The electromyography showed diffuse myopathic discharge, and muscle biopsy showed tubular aggregates. Genetic testing revealed five families with PPP carried *CACNA1S* (R1242S), *SCN4A* (R675Q, T704M), and *KCNJ2* (R218Q) respectively. The novel heterozygous R1242S mutation in *CACNA1S* caused a conformational change in the protein structure, and the amino acid of this mutation site was highly conserved among different species. *SCN4A* mutations led to two phenotypes of HypoPP2 and NormoPP. PPPs are autosomal dominant disorders of ion channel dysfunction characterized by episodic flaccid muscle weakness secondary to abnormal sarcolemmal excitability. PPPs are caused by mutations in skeletal muscle calcium channel Ca_V_1.1 gene (*CACNA1S*), sodium channel Na_V_1.4 gene (*SCN4A*), and potassium channels Kir2.1, Kir3.4 genes (*KCNJ2, KCNJ5*), including HypoPP1, HypoPP2, NormoPP, HyperPP, and ATS, which have significant clinical and genetic heterogeneity. Diagnosis is based on the characteristic clinical presentation then confirmed by genetic testing.

## Introduction

Periodic paralyses (PPs) are a group of skeletal muscle ion channelopathies characterized by episodic flaccid muscle weakness. The attacks may last hours to days or weeks, and often are associated with altered serum potassium (K^+^) levels. Patients usually complete recovery between the attacks, although some develop permanent fixed weakness later in life. A subset of PP patients accompany with cardiac arrhythmia or sudden cardiac death. PPs can be divided into primary periodic paralyses (PPPs) and secondary PPs by the etiology. Secondary PPs are common in hyperthyroidism, primary aldosteronism, renal tubular acidosis, and so on. Except for secondary PPs, PPPs can be suspected clinically.

PPPs are autosomal dominant disorders caused by mutations in genes encoding the skeletal muscle calcium channel (Ca_V_1.1), sodium channel (Na_V_1.4), and potassium channels (Kir2.1, Kir3.4). PPPs are classified as hyperkalemic periodic paralysis (HyperPP), normokalemic periodic paralysis (NormoPP), or hypokalemic periodic paralysis (HypoPP) based on serum K^+^ levels during paralytic attacks. Andersen-Tawil syndrome (ATS) is a rare PPP characterized by a triad of PP, cardiac arrhythmia, and skeletal and craniofacial anomalies, with high, low, or normal serum K^+^ levels.

HypoPP is caused by mutations in the alpha subunits of either the skeletal muscle L-type Ca_V_1.1 channel gene (*CACN1AS*, HypoPP1, 60%) or the skeletal muscle Na_V_1.4 channel gene (*SCN4A*, HypoPP2, 20%) [1]. HyperPP and NormoPP are associated with mutations in the Na_V_1.4 channel gene (*SCN4A)*. ATS is caused by mutations in the inward rectifying Kir 2.1, Kir3.4 channel genes (*KCNJ2, KCNJ5)* [2,3]. Unlike HypoPP, NormoPP and HyperPP, which are limited to mutations in channels expressed almost exclusively in skeletal muscle, the Kir 2.1, Kir3.4 channel mutations leading to ATS affect multiple tissues, and are associated with a highly variable phenotype of PP, cardiac arrhythmia, and skeletal and craniofacial anomalies [4].

PPPs have remarkable clinical and genetic heterogeneity. Here, we reviewed clinical manifestations, laboratory results, electrocardiogram (ECG), electromyography (EMG), muscle biopsy, and genetic analysis from five families with PPPs.

## Methods

### Patients

We retrospectively reviewed clinical and genetic features of five families with PPPs, whose main clinical manifestations were episodic flaccid weakness, with or without abnormal serum K^+^, and confirmed by genetic testing at the Third Hospital of Hebei Medical University in China between June 2016 and June 2020. All probands were examined by serum and urine K^+^, blood-gas analysis, aldosterone and renin-angiotensin levels on erect and supine positions, thyroid function, ultrasound analysis of the thyroid and adrenal glands to exclude secondary PPs, such as hyperthyroidism, renal tubular acidosis, primary aldosteronism and et al. Clinical, laboratory, ECG, EMG, muscle biopsy, and genetic data of these patients were collected. Written informed consent was obtained from each patient or their legal relatives before skeletal muscle biopsy and genetic testing. This study was approved by the ethics committee of the Third Hospital of Hebei Medical University and was conducted in accordance with the Declaration of Helsinki.

### Muscle biopsy and histochemistry stains

Open muscle biopsy was performed from the biceps brachii muscles. Biopsied skeletal muscles were snap frozen in isopentane that were cooled by liquid nitrogen and cut serially into 7 μm sections, preparing for histochemical staining and pathological analysis. Routine stains included hematoxylin and eosin (HE), modified Gomori trichrome (MGT), nicotinamide adenine dinucleotide-tetrazolium reductase (NADH-TR), succinic dehydrogenase (SDH), adenosine monophosphate deaminase (AMP), cytochrome c oxidase (CCO), acid phosphatase (ACID), adenosine triphospha-tase (ATPase) after incubation at pH 4.3, 4.5, and 10.1, periodic acid-Schiff (PAS), oil red O (ORO) and Sudan black B (SBB).

### Genetic testing

Genomic DNA was extracted from peripheral blood (obtained from all patients and some of their family members) with a DNA Kit. All the exon regions and adjacent intron regions (50bp) of 12 genes (*CACNA1A, CACNA1S, CLCN1, KCNA1, KCNE3, KCNJ16*, KCNJ18, *KCNJ2, KCNJ5, KCNQ1, SCN4A, CHRNB1*) related to ion channelopathies were sequenced by next-generation sequencing (NGS) (My Genostics, Beijing, China). The probable pathogenic mutations were then predicted using PolyPhen-2 (http://genetics.bwh.harvard.edu/pph2/), SIFT (http://sift.jcvi.org/), and MutationTaster (http://mutationtaster.org/). The functional verification of novel mutations was performed by conservative analysis of amino acid and tertiary structure prediction of protein. Sanger sequencing was used to verify the variants identified by NGS and to examine the available relatives of patients. The American College of Medical Genetics and Genomics (ACMG) standards were used to evaluate the pathogenicity [5].

## Results

### Clinical features

Detailed clinical, laboratory, ECG, and EMG data of the proband of five families with PPP are shown in Table 1. According to episodic flaccid weakness, serum K^+^ concentration during the attack, skeletal and craniofacial anomalies and cardiac arrhythmia in ECG, five families with PPPs were diagnosed with HypoPP (3/5), NormoPP (1/5), ATS (1/5).

**Table 1.** Clinical, laboratory, electrocardiogram, and electromyography data of five families with primary periodic paralysis

No.	Sex/Age/Onset age (y)	Family history	Preciptating factors	Clinical features							K^+^ level (mmol/L)	CK level (IU/L)	ECG	EMG	Muscle biopsy	Phenotype
				Myasthenia	Maximum weakness	Duraion	Frequency (Times)	Skeletal and craniofacial anomalies	Muscular volume	Muscular soreness						
1	M/50/10	+	high carbohydrate diet, resting post exercise, heat	Four limbs, paroxysmal	Bedridden	Several days	10–20/y	-	-	-	2.02–4.47	2836.8	-	-	degenerative or necrotic muscle fibers	HypoPP1
2	M/16/13	+	Fasting, resting post exercise	Four limbs, paroxysmal	Bedridden	Several days	6–9/y	-	-	+	3.3–4.5	351	-	/	-	HypoPP2
3	M/14/13.5	+	-	Two legs, paroxysmal	Unable to walk	Several days	3	-	-	+	3.3–4.58	1090	-	-	/	HypoPP2
4	M/34/10	+	Heavy exercise, being sedentary	Four limbs, paroxysmal	Bedridden	Several hours	10–15/y	-	Gastrocnemius hypertrophy	-	3.97–4.55	76.3	-	-	-	NormoPP
5	M/12/5	-	-	Four limbs, paroxysmal-permanent	Bedridden	Several hours	12–20/y	+	-	-	3.27–4.21	398	ventricular arrhythmias	myopathic changes	tubular aggregates	ATS

-, negative or normal. +, positive. /, unchecked. K^+^, potassium (reference interval, 3.5–5.5 mmol/L). CK, creatine kinase (reference interval, 50–310 IU/L). ECG, electrocardiogram. EMG, electromyography. M, male. y, year. HypoPP1, hypokalemic periodic paralysis type 1. HypoPP2, hypokalemic periodic paralysis type 2. NormoPP, normokalemic periodic paralysis. ATS, Andersen-Tawil syndrome.

**Figure 1.**
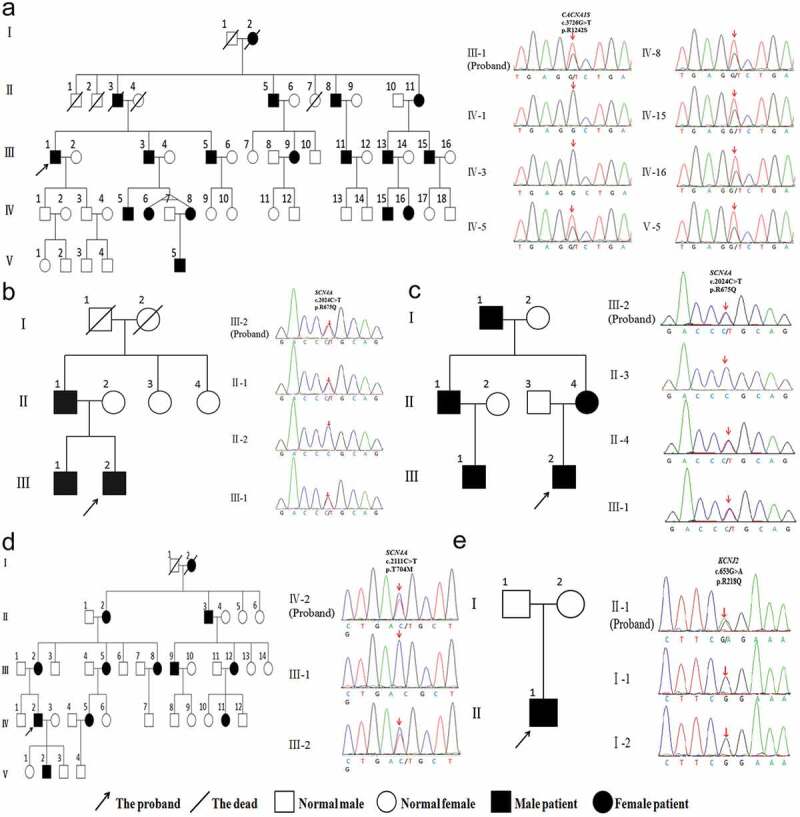
Pedigree and genetic analysis of five patients with primary periodic paralysis. (a) Patient 1, *CACNA1S* c.3726 G > T (p.R1242S) mutation. (b) Patient 2, *SCN4A* c.2024 C > T (p.R675Q) mutation. (c) Patient 3, *SCN4A* c.2024 G > A (p.R675Q) mutation. (d) Patient 4, *SCN4A* c.2111 C > T (p.T704M) mutation. (e) Patient 5, *KCNJ2* c.653 G > A (p.R218Q) mutation

**Figure 2.**
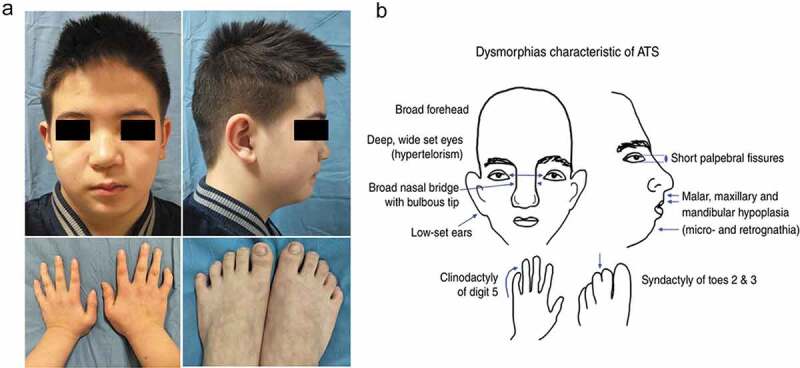
Skeletal-craniofacial features of the patient 5 with Andersen-Tawil Syndrome. (a) Widely spaced eyes, small mandible, low-set ears, and fifth-digit clinodactyly. (b) Dysmorphias characteristic of ATS, which can be found in Adams DS et al.(2016). DOI: 10.1113/JP271930

Five probands were male and presented with attacks of paroxysmal muscle weakness from childhood or adolescence. These attacks were generally precipitated by resting post exercise stress, heavy exercise, high carbohydrate diet, being sedentary, heat, and so on. They need to be bedridden because of severe muscle weakness, and usually recover completely between the attacks. Three probands (Patient 1–3) were diagnosed with HypoPP, whose serum K^+^ levels were lower at many times (reference interval (RI), 3.5–5.5 mmol/L) during the attacks. They complained of paroxysmal muscle weakness that lasted for several days, which often accompanied with muscle soreness, not muscle hypertrophy. Attacks appeared about several or dozens of times per year, which would be relieved after taking K^+^ supplement. Serum creatine kinase (CK) levels were elevated. Their family histories were positive (Figure 1(a–c)). One proband (Patient 4) was diagnosed with NormoPP, whose serum K^+^ levels were normal at many times during the attacks. He complained about paroxysmal muscle weakness that lasted for several hours, which accompanied with gastrocnemius hypertrophy, not muscle soreness. The family history was positive (Figure 1(d)). One proband (Patient5) showed skeletal and craniofacial anomalies with widely spaced eyes, small mandible, low-set ears, and fifth-digit clinodactyly, and no deformity of spine and extremities (Figure 2(a)). Cardiac arrhythmia was detected when he underwent a medical examination at the age of 5, but there were no symptoms of cardiopalmus or chest tightness. Ventricular extrasystoles were revealed using ECG. On 24 hr Holter examination frequent ventricular extrasystoles were detected. Echocardiography demonstrated a small amount of mitral regurgitation. He appeared in attacks of episodic flaccid weakness at the age of 8. The attacks lasted several days, and often were associated with low serum K^+^ levels. He developed permanent fixed myopathy at the age of 11, presenting with myopathic changes in EMG. There was no family history of sudden death, weakness, or dysmorphic features. ATS was suspected because of episodes of worsening limb weakness, his dysmorphic features, and presence of cardiac arrhythmia.

### Muscle pathological analysis

**Figure 3.**
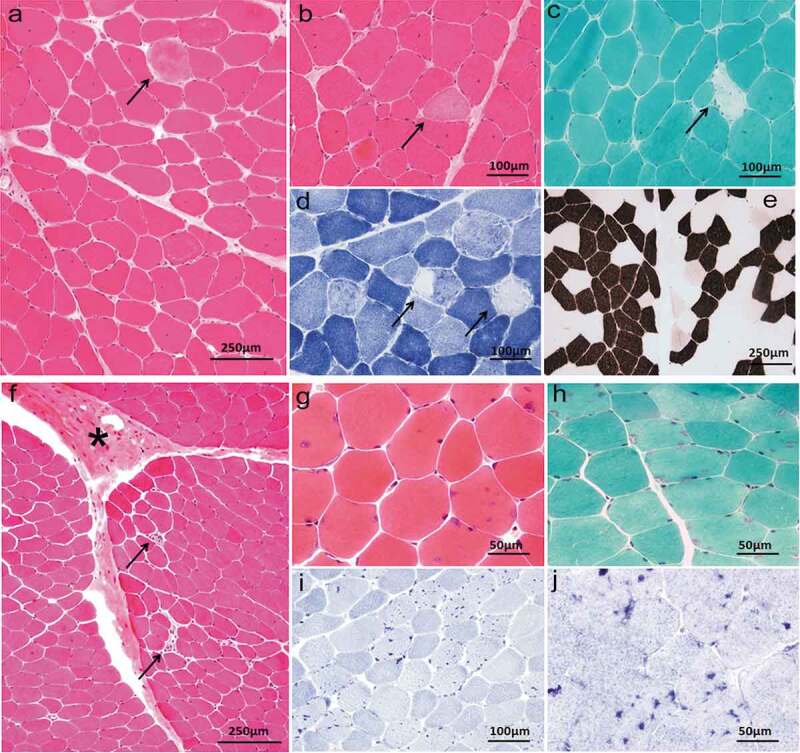
Muscle biopsy of patient 1,5. Patient 1, HE (a, b), MGT (c) and NADH-TR (d) demonstrated variation of fiber diameter, and scattered degenerative or necrotic muscle fibers (arrowhead). ATPase pH = 4.3 (e) demonstrated predominance of type 1 and type 2 fibers. Patient 5, HE (f, g), MGT (h), NADH-TR (i) and SDH (j) showed occasional degenerative or necrotic muscle fibers (arrowhead), connective tissue proliferation (asterisk) and lots of tubular aggregates (hyperchromatism)

Pathological analysis of skeletal muscle biopsy was performed in four probands (Patient 1,2,4,5). The muscle pathology of patient 1 (HypoPP) showed variation of fiber diameter and scattered degenerative or necrotic muscle fibers (Figure 3(a–d)). Myosin ATPase (pH = 4.3) staining (Figure 3(e)) showed mild predominance of type 1 and type 2 fibers. The muscle pathology of patient 5 (ATS) showed occasional degenerative or necrotic muscle fibers, connective tissue proliferation and lots of tubular aggregates (Figure 3(f–j)). The muscle pathology of the other two patients was almost normal and no special structure was found.

### Genetic analysis

**Figure 4.**
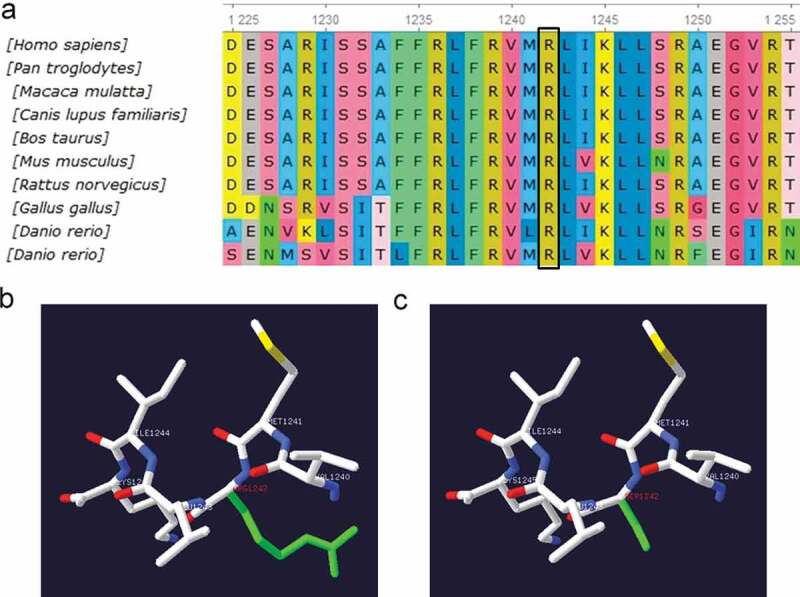
(a) Conservation of amino acid at the R1242S mutation site in different species. The amino acid sequence of R1242S mutation site was aligned with other species. (b) The tertiary structure prediction of protein in the wild type. (c) The tertiary structure prediction of protein in the mutant. R1242S mutation causes a conformational change in the protein structure (Green)

Sequencing showed that five probands with PPPs and some family members had carried three different gene mutations, including *CACNA1S, SCN4A,* and *KCNJ2*, which were heterozygous missense mutations (Figure 1). *SCN4A*(p.R675Q, p.T704M) and *KCNJ2* (p.R218Q) had been reported in the literature [2,6–12]. Among them, *KCNJ2*(p.R218Q) mutation was spontaneous, which was found in the proband but not his parents. A novel heterozygous R1242S mutation in *CACNA1S* gene was found in the proband with HypoPP and some family members. The amino acid at the R1242S mutation site was highly conserved among different species (Figure 4(a)). R1242S mutation caused a conformational change in the protein structure (Figure 4(b,c)). The pathogenicity of gene mutations was judged by the ACMG standards [5], and all of them were pathogenic or likely pathogenic. Genetic results are listed in Table 2.

**Table 2.** The gene mutation information of five families with primary periodic paralysis

Patient no.	Phenotype	Gene	Coding channel	Exon/haplotype	Nucleotide change	Protein change	Structural position	Variant classification (ACMG)	Variant source
1	HypoPP1	*CACNA1S*	Ca_V_1.1	30/het	c.3726 G > T	p.R1242S	DⅣS4	Likely pathogenic	Father
2	HypoPP2	*SCN4A*	Na_V_1.4	13/het	c.2024 C > T	p.R675Q	DII S4	Likely pathogenic	Father
3	HypoPP2	*SCN4A*	Na_V_1.4	13/het	c.2024 C > T	p.R675Q	DII S4	Likely pathogenic	Mother
4	NormoPP	*SCN4A*	Na_V_1.4	13/het	c.2111 C > T	p.T704M	DII S5	Likely pathogenic	Mother
5	ATS	*KCNJ2*	Kir2.1	2/het	c.653 G > A	p.R218Q	C-terminal	Pathogenic	Spontaneous mutation

ACMG, the American College of Medical Genetics and Genomics. HypoPP1, hypokalemic periodic paralysis type 1. HypoPP2, hypokalemic periodic paralysis type 2. NormoPP, normokalemic periodic paralysis. ATS, Andersen-Tawil syndrome. C-terminal, carboxy-terminal.

## Discussion

**Figure 5.**
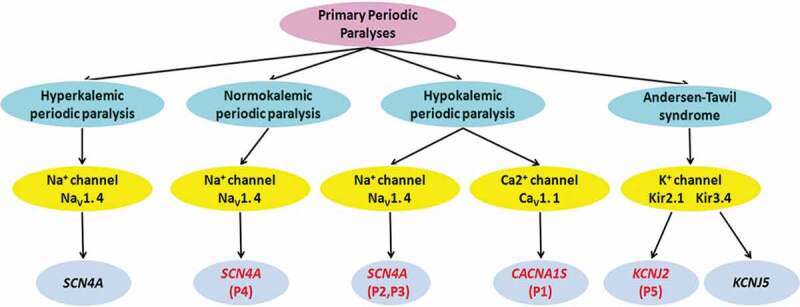
Clinical classification of primary periodic paralyses and the associated gene defects. P1-5:Patient 1–5

PPPs are a group of autosomal-dominant genetic ion channelopathies caused by mutations in skeletal muscle Ca_V_1.1 channel gene (*CACNA1S*), Na_V_1.4 channel gene (*SCN4A*), Kir 2.1 and Kir3.4 channel genes (*KCNJ2, KCNJ5*), including HypoPP1, HypoPP2, NormoPP, HyperPP, and ATS, which have remarkable clinical and genetic heterogeneity. There are five families with PPPs, whose main clinical manifestations were episodic flaccid weakness, with or without abnormal serum K^+^, and confirmed by genetic testing, including HypoPP1 (*CACNA1S*, 1/5), HypoPP2 (*SCN4A*, 2/5), NormoPP (*SCN4A*, 1/5) and ATS (*KCNJ2*, 1/5) in this study (Figure 5).

### Clinical phenotypes

The basic clinical manifestations of five families were consistent with PPP, presenting with paroxysmal muscle weakness, with or without abnormal serum K^+^. PPPs are autosomal dominant or sporadic diseases that are more prevalent in men from childhood or adolescence, which may be associated with sex hormones [13]. The predisposing factors of PPPs are generally precipitated by resting post exercise, heavy exercise, high carbohydrate diet, fasting, prolonged rest, heat or cold, stress, alcohol, and so on [4,14]. Attacks of weakness in NormoPP/HyperPP tend to be shorter in duration than attacks in HypoPP, and gastrocnemius hypertrophy is more commonly found in NormoPP/HyperPP [4,15]. Two patients with HypoPP2 caused by R675Q mutation were accompanied by muscular soreness, indicating that muscular soreness may be closely related to the mutation gene and site. One patient caused by R1242S mutation in *CACNA1S* gene exhibited the characteristic clinical and laboratory features of HypoPP1, including hypokalemia during attacks and effective remission of weakness after supplying K^+^. A study reported that one family with R1242G mutation present with NormoPP and transient compartment-like syndrome, which were not found in other reports with mutation in *CACNA1S* gene and our family [16]. In our study, the serum CK levels were elevated in HypoPP. Hypokalaemia might impair the energy supply to the muscle cells, which would increase the permeability of the sarcolemma, and result in a rise in serum CK [17].

ATS is a rare ion channel disorder characterized by a triad of PP, cardiac abnormalities, and skeletal and craniofacial anomalies. These episodes of PP are more frequently associated with hypokalemia than with hyperkalemia or normokalemia. In this study, episodes of PP are associated with hypokalemia and permanent proximal weakness develops later in the patient with AST. Permanent weakness often develops before or after the classical episodic weakness in related literature [18–20]. Ventricular arrhythmia is the most common cardiac abnormality in patients with AST. Other manifestations include prolonged QT interval, bidirectional ventricular tachycardia, prominent U waves, and cardiac arrest. Skeletal and craniofacial anomalies include low-set ears, widely spaced eyes, small mandible, fifth-digit clinodactyly, and syndactyly of digits 2–3. In addition, there may be broad forehead, wide bridge of nose, abnormal or absence of the lateral incisors, high arch and palate, cleft palate, relative microcephaly, small hands and feet, scoliosis, and so on (Figure 2(b)) [2,19,21]. In this study, the patient had typical skeletal and craniofacial anomalies, such as low-set ears, widely spaced eyes, small mandible and fifth-digit clinodactyly (Figure 2(a)). Not all ATS patients present with the full triad of symptoms. Moreover, the most common dysmorphic features are usually present, but may be very mild and not always easily recognized as part of the syndrome [4]. This patient with ATS is treated with ventricular arrhythmia as the first symptom. ATS occurs with a high degree of phenotypic variability, rendering diagnosis very difficult. Genetic testing may confirm the clinical diagnosis of ATS by revealing the presence of a pathogenic mutation.

### Muscle pathology

The typical muscle pathological changes of PPPs are vacuoles and tubular aggregates under the membrane or in muscle fibers, which may be caused by dilatation of sarcoplasmic reticulum and abnormal protein deposition [22–24]. Tubular aggregates are more commonly seen in ATS [2,25,26]. In addition, variation in diameter of muscle fibers, fiber splitting, internal nuclei, myofiber necrosis, and other pathological changes can be observed [24]. Patients with fixed weakness usually have nonspecific myopathic changes, with or without vacuolar change and tubular aggregates [24]. In our study, degenerative or necrotic muscle fibers and mild predominance of type 1 and 2 fibers were found in a patient with HypoPP1 (Figure 3). Typical tubular aggregates were seen in the ATS patient with permanent weakness (Figure 3). Due to the lack of specific pathological characteristics, gene analysis can be carried out directly after clinical diagnosis. The muscle biopsy in patients with PPPs is significant in terms of a better understanding of the pathophysiology.

### Genetics

PPPs can be caused by mutations in genes encoding the skeletal muscle membrane channel proteins, which have a high degree of genetic heterogeneity. HypoPP1 is caused by mutations of Ca_V_1.1 channel gene (*CACNA1S*), while HypoPP2, NormoPP, and HyperPP are caused by mutations of Na_V_1.4 channel gene (*SCN4A*). ATS is caused by mutations of Kir2.1 and Kir3.4 channel genes (*KCNJ2, KCNJ5*) (Figure 5).

A novel heterozygous mutation R1242S is localized at the S4 segment of domain IV in the alpha subunit of the L-type skeletal muscle voltage-gated Ca_V_1.1 channel encoded by *CACNA1S* gene, caused the third positively charged arginine replaced by hydrophobic amino acid residue. Among the HypoPP1 mutations identified so far, most consist in substitutions at positively charged arginine residues in S4 segments, which could lead to the formation of a gating pore current at hyperpolarized potentials, including R528, R897, R900, R1239, and so on [27,28]. The gating pore current causes susceptibility to paradoxical depolarization during attacks. The depolarization of sarcolemma inactivates Na_V_1.4 channels to cause periodic paralysis [27,28]. As a neighboring site of R1239, the R1242S mutation may also generate a gating pore current, which needs to be proven in the future. The R1242S mutation would have an important effect on the structure and function of the Ca_V_1.1 channel protein, considering the fact that this residue is highly conserved among different species (Figure 4(a)) and mutation causes a conformational change in the protein structure (Figure 4(b,c)).

R675Q mutation is located at the S4 segment of domain II in the alpha subunit of the skeletal muscle voltage-gated Na_V_1.4 channel encoded by *SCN4A* gene, which causes the third positively charged arginine replaced by hydrophobic amino acid residue, leading to the formation of abnormal gating pore current. The gating pore current causes susceptibility to paradoxical depolarization during attacks. The depolarization of sarcolemma leads to inactivation of Na_V_1.4 channels to cause periodic paralysis [29,30]. It has been reported that R675Q mutation can cause HypoPP2 in Chinese patients [6], but this mutation is more common in NormoPP [8,9] and can also be seen in HyperPP [31]. T704M mutation in the S5 segment of domain II can result in impaired fast or slow inactivation of Na_V_1.4 channel, and, therefore, persistent increased sodium current and sarcolemmal depolarization, which can result in the symptom of paralysis [32,33]. It has been reported that T704M mutation can cause NormoPP [7,10].

*KCNJ2* encodes the Kir2.1 inward rectifier potassium channel, which is mainly expressed in skeletal muscle and cardiac myocytes. R218Q mutation is located at the carboxy-terminal (C-terminal) region of Kir2.1 channel protein, which reduces the amount of inward rectifier current, decelerates the action potential repolarization, and prolongs the action potential duration, thereby leading to ventricular arrhythmias associated with ATS [34]. Reduced Kir2.1 channel function in skeletal muscle may cause the resting membrane depolarization, leading to failure of action potential propagation and flaccid paralysis [26]. It has been reported that R218Q mutation can cause ATS [2,11,12]. Mutations in Kir2.1 reduce the amount of inward rectifier current and might hamper the adequate functioning of osteoclasts, thereby interfering with the balance between bone formation and resorption, which might explain the mild skeletal anomalies in patients with ATS [35]. Kir2.1 is expressed during the earliest stages of craniofacial development. The anomalies in Kir2.1 lead to misexpression of developmentally regulated craniofacial genes, and contribute to abnormalities in craniofacial development [2,19].

## Conclusion

PPPs are autosomal dominant disorders of ion channel dysfunction characterized by episodic flaccid weakness secondary to abnormal sarcolemmal excitability, which are caused by mutations in skeletal muscle Ca_V_1.1 channel gene (*CACNA1S*), Na_V_1.4 channel gene (*SCN4A*), Kir 2.1 and Kir3.4 channel genes (*KCNJ2, KCNJ5*). PPPs are classified as HyperPP, NormoPP, or HypoPP based on the concomitant serum K^+^ levels. ATS is different from the other phenotypes of PPPs, characterized by a highly variable phenotype of PP, ventricular arrhythmia, and distinctive facial and skeletal anomalies, associated with high, low, or normal serum K^+^ levels. Diagnosis is based on the characteristic clinical presentation then confirmed by genetic testing.
